# Successful treatment of peritoneal dialysis for two patients with refractory nephrotic syndrome and acute kidney injury: a case report

**DOI:** 10.3389/fmed.2023.1263780

**Published:** 2023-10-18

**Authors:** Le Yu, Shuiqin Cheng, Man Zhang, Tingting Zhou, Yunmin Chen, Zhihong Zhang, Yusheng Yu

**Affiliations:** National Clinical Research Center for Kidney Disease, Jinling Hospital, Nanjing, China

**Keywords:** peritoneal dialysis, refractory nephrotic syndrome, acute renal injury, renal supportive therapy, diuretic resistance

## Abstract

Two patients with refractory nephrotic syndrome were treated with peritoneal dialysis (PD) for diuretic resistance, anasarca and acute kidney injury. Following PD, their fluid overload was promptly alleviated, accompanied by an increase in urine volume and an improvement in renal function. PD as an adjuvant approach enabled them to resume corticosteroids and immunosuppressive agents. Eventually, both patients could be withdrawn from PD and achieved remission of proteinuria.

## 1. Introduction

Refractory nephrotic syndrome (RNS) is defined as: frequent relapses or infrequent relapses with significant steroid toxicity; and failure of 2 or more steroid-sparing agents (1, 2). In Japan, RNS represents a pathological condition in which resistance to steroids and other immunosuppressive agents persists for at least 6 months, and 10 to 12% of idiopathic nephrotic syndrome (NS) fall into this category (3). Among adults with minimal-change disease (MCD), around 10–20% are steroid-resistant nephrotic syndrome (SRNS) (4). Repeated biopsies showed a substantial proportion of such patients progressing to focal segmental glomerulosclerosis (FSGS), which is associated with an unfavorable prognosis (5). The incidence of SRNS in children exceeds 40% (6). Furthermore, 20–40% of children exhibit resistance not only to steroids but also to other immunosuppressive agents (7). Merely 20% of patients achieved complete remission, 33% had partial remission, and 47% did not achieve remission with all prescribed therapies (8). Ten-year End-Stage Renal Disease (ESRD)-free survival rates were 43, 72, and 94% for children displaying intensified immunosuppression resistance, partial remission, and complete remission, respectively (9). This corresponds to the data in adults with FSGS (10, 11). Complications such as acute kidney injury (AKI), infection and thrombosis may recur during the course of RNS (12). Herein, we present two cases of AKI complicating RNS that were successfully managed with peritoneal dialysis (PD). We integrated the concept of kidney protection and support throughout the treatment, and ultimately achieved favorable clinical outcome.

## 2. Case description

### 2.1. Case 1

A 23-year-old male was admitted to a local hospital with complaints of edema and reduced urine volume. Urinalysis showed 4+ of proteinuria with mild hematuria. Laboratory examinations revealed serum albumin of 10.1 g/L and serum creatinine of 141umol/L, indicating NS with mild renal insufficiency. He was treated with Methylprednisolone 50 mg/d, leflunomide, low-molecular-weight heparin, and furosemide. Ten days later, the patient developed a low-grade fever and lumbar skin infection, along with chest tightness. A computerized tomography scan showed lung inflammation and massive effusion in the chest and abdominal cavity. Antibiotics were administered, along with human albumin infusions and intravenous furosemide. Then his serum creatinine rose to 360umol/L, and there was no response to diuretics. The patient’s edema deteriorated progressively, leading to the commencement of prolonged intermittent hemodialysis (IHD) (8 h every time) 1 month after the onset.

Then the patient was referred to our hospital and received a reduced dose of methylprednisolone. His urinary protein excretion was 28.17 g/d, and his serum albumin and creatinine were 17.2 g/L and 2.98 mg/dL, respectively. He tested negative for antinuclear antibody, anti-double-stranded DNA antibody, anti-phospholipase A2 receptor antibodies, as well as viral infections. Ultrasound showed enlarged kidneys (left: 127 × 61 × 63 mm; right: 130 × 66 × 65 mm) with hyperechogenicity of renal parenchyma and blurred boundary of cortex and medulla. Kidney biopsy showed IgA nephropathy with MEST-C score M0, E1, S1, T0, C1.

Due to rapidly progressive glomerulonephritis, the patient received intravenous methylprednisolone (0.5 g daily for 3 days) and cyclophosphamide (0.6 g once), followed by oral methylprednisolone at a dosage of 24 mg/d. He also underwent prolonged IHD three times per week due to severe azotemia. After 2 weeks, his serum creatinine improved to 2.3 mg/dL. IHD was discontinued, and the patient prescribed tacrolimus, while the dosage of methylprednisolone was tapered. However, He continued to experience persistent massive proteinuria, elevated markers of renal tubular injury, fluctuating serum creatinine around 3 mg/dL, urea nitrogen between 71.75 and 84.32 mg/dL, and refractory hypernatremia (sodium of 158–162.7 mmol/L, chloride of 125.1–131.5 mmol/L), which necessitated discontinuation of tacrolimus. Despite treatment with furosemide, calcium channel blockers, α-blockers, and β-blockers, blood pressure control was still difficult. Moreover, Despite the use of diuretics, his urine output remained low (700 mL/d) and body weight increased by 4 kg in 1 week in the absence of IHD. Consequently, He developed abdominal distension, chest tightness, and cough due to a significant amount of effusion in the serous cavities, requiring IHD again.

The patient was readmitted with severe edema and abdominal distension. Physical examination revealed abdominal bulging, positive shifting dullness, and marked edema. A PD catheter was inserted 6 months after the onset of NS., and approximately 500 mL of ascites was drained during the intraoperative procedure. PD treatment commenced the following day, and daily drainage of ascites was performed to alleviate intra-abdominal pressure. In addition, angiotensin-converting enzyme inhibitor (ACEI) was initiated after PD, while all diuretics and other antihypertensive drugs were discontinued. For address malnutrition, A dietary plan was devised. Upon discharge, the patient’s dialysis prescription comprised two manual daytime dwells of 2 liters, 1.5% glucose, for 4 h each. More than a month after PD, the patient’s urine output increased to approximately 1,500 mL/d, the edema subsided, and the appetite improved. Then we attempted to increase the dosage of steroids and reintroduce tacrolimus. Follow-up showed that the patient’s urine output remained around 2,000 mL/d, with no signs of edema, normal electrolytes levels, and a decrease in serum creatinine to approximately 1.6 mg/dL and stabilized.

**Figure 1:**
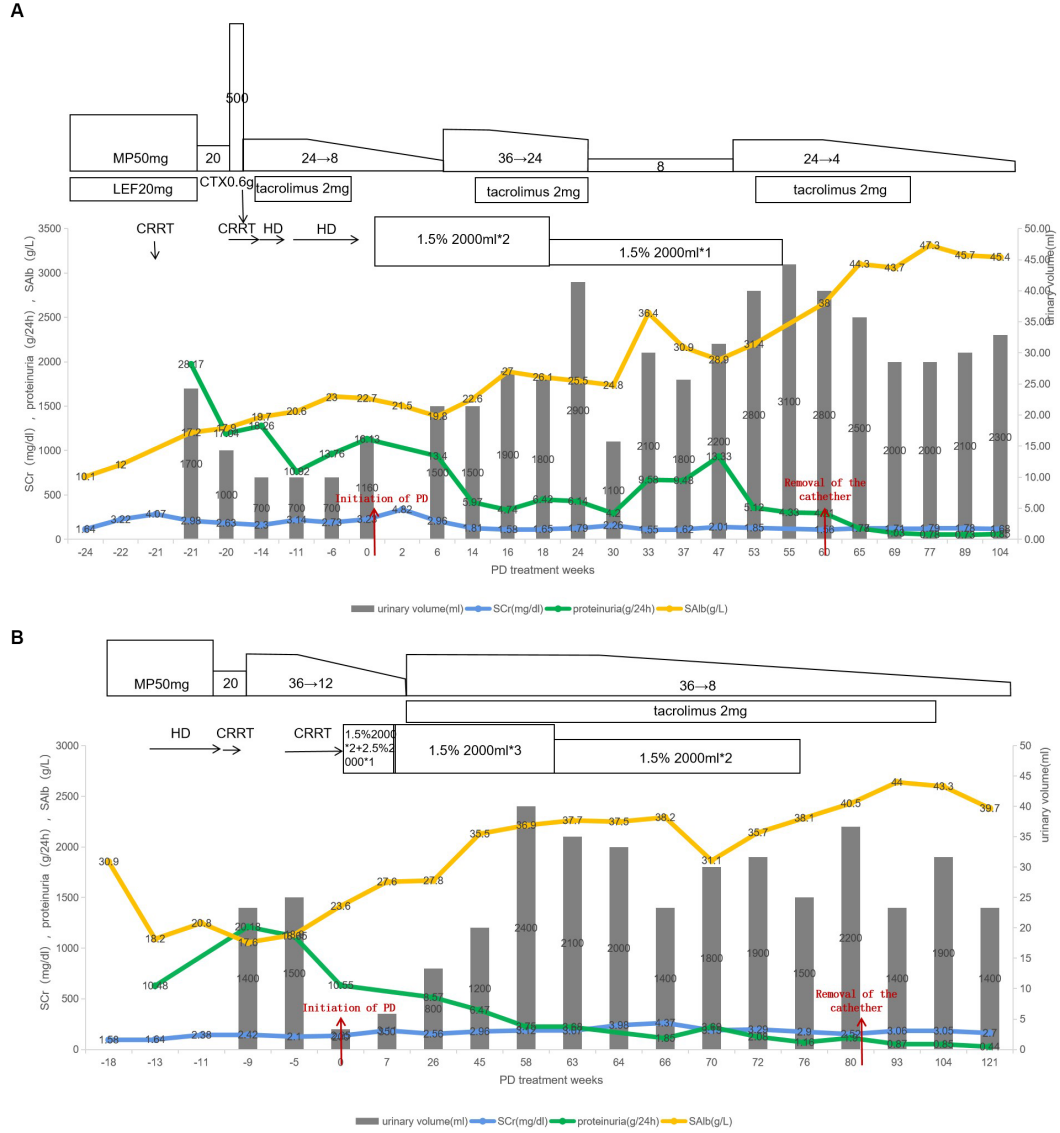
**(A,B)** Showed the treatment process of patient A and patient B, respectively. CRRT, continuous renal replacement therapy; HD, hemodialysis; PD, peritoneal dialysis; MP, methylprednisolone; CTX, cyclophosphamide; SAlb, serum albumin.

But then, the patient suffered skin injuries on both legs after a fall, leading to the formation of several abscesses in the left ankle and leg muscles. A culture of the pus identified a *Staphylococcus aureus* infection. Concurrently, pneumocystis carinii pneumonia and cytomegalovirus infections were detected through next-generation sequencing technology. The patient also experienced hyperglycemia. To address these issues, he discontinued tacrolimus and received anti-infective therapy, and local treatment with debridement. Low-dose corticosteroids, ACEI, daglitazone, rivaroxaban, and other medications were administered as well. Subsequently, the patient encountered several other complications, including retinal vein occlusion in the left eye, gastrointestinal bleeding, and bone infarction. Luckily, these complications improved with symptomatic treatment and the patient’s nutritional status gradually improved. Despite persistent massive proteinuria, the patient maintained a urine volume exceeding 2,000 mL/d. After excluding infection, the methylprednisolone dosage was increased, and tacrolimus was resumed. Then the urinary protein excretion gradually decreased, the serum albumin returned to the normal range, and renal function steadily improved, culminating in the successful removal of the PD catheter at 84 weeks after the onset of NS. Follow-up 6 months after discontinuing PD revealed that the patient’s urinary protein excretion was 0.78 g/d, urine volume was 2000 mL/d, serum albumin was 47.3 g/L, and serum creatinine was 1.79 mg/dL (Figure 1A).

### 2.2. Case 2

A 24-year-old female teacher developed edema in her lower limbs following an upper respiratory tract infection. A urinalysis performed at a local hospital revealed significant proteinuria (4+) and hematuria (1+). The patient’s serum albumin was 30.9 g/L, and serum creatinine was 139.6 umol/L. A kidney biopsy confirmed the diagnosis of minimal change disease. However, despite receiving full-dose steroids, the patient’s condition did not improve. Four weeks after the onset of NS, she underwent prolonged IHD due to oliguria and the presence of massive pleural and abdominal effusion.

Six weeks after the onset of NS, the patient was admitted to our department with an 8 kg weight gain compared to her pre-illness weight. The physical examination revealed Cushingoid features and severe generalized edema. Laboratory examinations indicated urinary protein excretion of 18.67 g/24 h, serum albumin of 21.10 g/L, serum creatinine of 2.47 mg/dL, along with hyperlipidemia and anemia. Autoantibody tests yielded negative results. CT scans revealed significant fluid accumulation in the chest and abdominal cavities, and b-ultrasound imaging displayed enlarged kidneys (left: 126 × 53 × 63 mm; right: 121 × 53 × 60 mm). The steroid dosage was reduced, while albumin infusion, prophylactic anticoagulation, and diuresis were continued. Subsequently, the patient’s renal function remained stable, and urine volume recovered to 1,300 mL/d. IHD was discontinued, and the venous catheter was removed. Prior to discharge, the methylprednisolone dosage was increased to 36 mg/d. Unfortunately, the patient experienced herpes zoster and recurrent skin lesions, and exacerbated edema. Consequently, she required recharging and underwent another round of IHD after reinsertion of the vein catheter.

Then her urine output decreased to 200 mL/d, and she suffered from symptoms of anorexia and vomiting. Given the absence of remission in NS and the presence of significant gastrointestinal symptoms, discontinuation of dialysis was not a viable option. At 18 weeks after the onset of NS, she underwent the insertion of a PD catheter and initiated PD treatment. Before removing the jugular vein catheter, an ultrasound examination revealed a thrombus at the insertion site. Anticoagulant therapy was continued after the catheter removal. The initial PD prescription consisted of two dwells of 2 liters with 1.5% glucose and one dwell of 2 liters with 2.5% glucose, each lasting for a duration of 4 h. The dosage of diuretics was gradually reduced, while protein intake was augmented to address the malnutrition.

After 1 month of PD, there was a gradual amelioration in both edema and gastrointestinal discomfort. Consequently, the PD prescription was adjusted to three manual daytime dwells of 2 liters with 1.5% glucose. Given her stable overall condition and the absence of infection, the steroid dosage was increased and tacrolimus was added. Subsequent laboratory examinations revealed a gradual decrease in urinary protein, a progressive increase in serum albumin, and an improvement in urine output.

After 13 months of PD treatment, the patient’s total Kt/V urea was 2.43 and creatinine clearance was 190.73 L/week/1.73 m2. Consequently, her PD prescription was decreased to two dwells of 2 liters with 1.5% glucose. After 76 weeks of PD treatment, her urinary protein excretion reduced to 1.16 g/d, serum albumin increased to 38.1 g/L, and serum creatinine decreased to 2.9 mg/dL. Subsequently, the PD treatment was discontinued. After 1 month without any signs of edema and with stable kidney function, the PD catheter was removed (Figure 1B).

## 3. Discussion

Roughly 30% of patients with NS experience complications involving AKI (13). Infection, exposure to nephrotoxic medication, and steroid resistance are the primary risk factors for AKI in patients with NS (14). More severe AKI is correlated with a diminished rate of complete remission and an extended time for achieving remission in patients with NS (13). Smith et al. (15) analyzed 75 cases of NS with AKI across 16 literatures, of which 64 cases of MCD(85%), 6 cases of FSGS, 4 cases of mesangial proliferation, and 1 case of focal proliferation.

We frequently observe that patients with combined RNS and AKI, who undergo extracorporeal ultrafiltration or IHD, often tend to display a rapid loss of renal function. Sporadic reports have explored the utilization of PD for managing patients with RNS and AKI. Barman et al. (16) employed PD in a pediatric case. Prior to PD treatment, the child exhibited diuretic resistance and significant edema. Following PD, there was a reduction in volume overload, recovery of AKI, an increase in urine output, and restoration of diuretic efficacy. Another case involved a diabetic patient who presented with acute oliguric renal failure and NS,. Following 8 months of PD treatment, the patient’s renal function improved and urinary volume returned to normal ranges, leading to the discontinuation of PD (17). In addition to the conventional glucose PD solution, Morimoto et al. (18, 19) reported successful treatment of three RNS patients using icodextrin. Whereas, there remains a paucity of articles that offer a comparison between PD and HD for treating patients with NS and AKI. Sakarcan et al. (20) documented four children with primary NS complicated with AKI, of whom three underwent HD and one underwent PD. The children undergoing HD ceased dialysis within 9 days and recovered from AKI within 20 days. In contrast, the dialysis duration and renal function recovery in the child undergoing was 1 year. However, after discontinuing dialysis, the urine volume of child undergoing PD is relatively higher.

Compared to HD, PD offers a more physiological dialysis mechanism, better hemodynamic tolerance, and less inflammation due to no exposure to synthetic membranes. These integrated factors potentially contribute to renal recovery (21, 22). Nonetheless, PD is associated with a lower volume of fluid removal compared to extracorporeal modalities, implying that PD might not be an optimal choice when the primary goal of renal replacement therapy is to eliminate fluid overload (23).

However, in our cases, ultrafiltration of PD can compensate for inadequate urine volume and fulfill daily fluid output requirements of the patients. The gentle ultrafiltration process inherent to PD mitigates hemodynamic disturbances, thus preserving residual kidney function in individuals with NS and insufficient effective blood volume (24). The sodium concentration in the peritoneal dialysate, measuring 132 mmol/L, is lower than the serum sodium in patients, facilitating the elimination of excess sodium. In addition, the osmotic pressure of the peritoneal dialysate exceeds that of the patient’s plasma. Therefore, PD acts akin to a loop diuretic, but without the side effects of pharmacological diuresis. Both patients experienced a prompt resolution of their volume overload issues following PD. Patient A achieved a urine volume recovery of over 1,500 mL/d, which was sustained long-term after discontinuation of diuretics within 1.5 months of PD. Despite patient B experienced a delayed recovery, her urine output gradually increased to 800 mL/d after initiating PD and continued to rise, surpassing 2,000 mL/d after 1 year of PD.

Additionally, it has long been held that PD is unsuitable for patients with NS because of additional albumin losses owing to PD (25, 26). However, research findings suggest that serum albumin is only approximately 0.3 g/dL lower in PD (27). We postulate that patients undergoing PD experience improvements in both intestinal edema and azotemia, which consequently contribute to the amelioration of their appetite. PD allows them to increase protein intake and ensures adequate caloric intake, thereby maintaining energy and nitrogen balance and effectively reversing malnutrition.

Putting aside the benefits of stable ultrafiltration and correcting malnutrition provided by PD mentioned above. Moreover, most of the patients with RNS demonstrate hypercoagulability. PD exerts minimal impact on coagulation dynamics, reducing venous catheterization thrombosis. Last but not least, PD is performed by patients themselves at home. This arrangement not only alleviates the burden of frequent hospital visits on patients but also mitigates the likelihood of opportunistic infections. Furthermore, it is a more cost-effective alternative to extracorporeal ultrafiltration.

## 4. Conclusion

In conclusion, we report the successful application of PD in two patients with RNS and AKI, which has highlighted the crucial role of PD in managing RNS. PD contributes to preserving residual renal function (RRF), improving patients’ nutritional status, and providing guarantees for further immunosuppressive therapy of RNS. We firmly believe that PD can function not only as a replacement method but also as an adjuvant approach.

## Data availability statement

The original contributions presented in the study are included in the article/Supplementary material, further inquiries can be directed to the corresponding author.

## Ethics statement

The studies involving humans were approved by the local Ethics Committee of Jinling Hospital. The studies were conducted in accordance with the local legislation and institutional requirements. The participants provided their written informed consent to participate in this study. Written informed consent was obtained from the individual(s) for the publication of any potentially identifiable images or data included in this article.

## Author contributions

LY: Conceptualization, Data curation, Formal analysis, Investigation, Methodology, Software, Visualization, Writing – original draft. SC: Formal analysis, Investigation, Methodology, Writing – original draft, Conceptualization, Data curation. MZ: Conceptualization, Data curation, Investigation, Software, Writing – original draft. TZ: Resources, Supervision, Validation, Writing – review & editing. YC: Project administration, Software, Writing – review & editing. ZZ: Resources, Supervision, Writing – review & editing. YY: Conceptualization, Funding acquisition, Project administration, Supervision, Writing – review & editing.

## References

[1] Sinha A Bagga A Banerjee S Mishra K Mehta A Agarwal I. Steroid sensitive nephrotic syndrome: revised guidelines. Indian Pediatr. (2021) 58:461–81. doi: 10.1007/s13312-021-2217-3, PMID: 33742610 PMC8139225

[2] Taşdemir M Canpolat N Yıldız N Özçelik G Benzer M Saygılı SK. Rituximab treatment for difficult-to-treat nephrotic syndrome in children: a multicenter, retrospective study. Turk J Med Sci. (2021) 51:1781–90. doi: 10.3906/sag-2012-297, PMID: 33581711 PMC8569731

[3] Saito T. Refractory nephrotic syndrome. Nihon Rinsho. (2004) 62:1794–9. PMID: 15500120

[4] Nolasco F Cameron JS Heywood EF Hicks J Ogg C Williams DG. Adult-onset minimal change nephrotic syndrome: a long-term follow-up. Kidney Int. (1986) 29:1215–23. doi: 10.1038/ki.1986.130, PMID: 3747335

[5] Meyrier A Noël LH Auriche P Callard P. Long-term renal tolerance of cyclosporin a treatment in adult idiopathic nephrotic syndrome. Collaborative Group of the Société de Néphrologie. Kidney Int. (1994) 45:1446–56. doi: 10.1038/ki.1994.189, PMID: 8072258

[6] Kim JS Bellew CA Silverstein DM Aviles DH Boineau FG Vehaskari VM. High incidence of initial and late steroid resistance in childhood nephrotic syndrome. Kidney Int. (2005) 68:1275–81. doi: 10.1111/j.1523-1755.2005.00524.x, PMID: 16105061

[7] Gibson KL Hansrivijit P Ferris ME. Emerging agents for the management of nephrotic syndrome: progress to date. Paediatr Drugs. (2016) 18:25–9. doi: 10.1007/s40272-015-0148-y, PMID: 26645400

[8] Gipson DS Chin H Presler TP Jennette C Ferris ME Massengill S. Differential risk of remission and ESRD in childhood FSGS. Pediatr Nephrol. (2006) 21:344–9. doi: 10.1007/s00467-005-2097-0 16395603

[9] Trautmann A Schnaidt S Lipska-Ziętkiewicz BS Bodria M Ozaltin F Emma F. Long-term outcome of steroid-resistant nephrotic syndrome in children. J Am Soc Nephrol. (2017) 28:3055–65. doi: 10.1681/asn.2016101121, PMID: 28566477 PMC5619960

[10] Troyanov S Wall CA Miller JA Scholey JW Cattran DC. Focal and segmental glomerulosclerosis: definition and relevance of a partial remission. J Am Soc Nephrol. (2005) 16:1061–8. doi: 10.1681/asn.2004070593 15716334

[11] Cattran D. Management of membranous nephropathy: when and what for treatment. J Am Soc Nephrol. (2005) 16:1188–94. doi: 10.1681/asn.2005010028 15800117

[12] Tullus K Webb H Bagga A. Management of steroid-resistant nephrotic syndrome in children and adolescents. Lancet Child Adolesc Health. (2018) 2:880–90. doi: 10.1016/s2352-4642(18)30283-9 30342869

[13] Chen T Lv Y Lin F Zhu J. Acute kidney injury in adult idiopathic nephrotic syndrome. Ren Fail. (2011) 33:144–9. doi: 10.3109/0886022x.2011.553301 21332335

[14] Meyrier A Niaudet P. Acute kidney injury complicating nephrotic syndrome of minimal change disease. Kidney Int. (2018) 94:861–9. doi: 10.1016/j.kint.2018.04.024 29980292

[15] Smith JD Hayslett JP. Reversible renal failure in the nephrotic syndrome. Am J Kidney Dis. (1992) 19:201–13. doi: 10.1016/s0272-6386(13)80001-7 1553965

[16] Barman H Sirie R Duwarah SG. Effective ultrafiltration with acute peritoneal dialysis in a child with diuretic-resistant nephrotic edema. Saudi J Kidney Dis Transpl. (2015) 26:743–6. doi: 10.4103/1319-2442.160197 26178548

[17] Avilés-Santa L Alpern R Raskin P. Reversible acute renal failure and nephrotic syndrome in a type 1 diabetic patient. J Diabetes Complicat. (2002) 16:249–54. doi: 10.1016/s1056-8727(01)00221-5 12015196

[18] Morimoto S Takahashi N Kikuchi S Yamahara H Imada T Kohno K. Management of patients with recurrent nephrosis and intractable edema by intraperitoneal instillation of icodextrin solution. Perit Dial Int. (2008) 28:559–62. doi: 10.1177/089686080802800527, PMID: 18708557

[19] Morimoto S Takahashi N Someya K Morita T Jo F Toyoda N. A patient with refractory nephrotic syndrome withdrawn from peritoneal dialysis. Clin Exp Nephrol. (2010) 14:363–6. doi: 10.1007/s10157-010-0271-6, PMID: 20186457

[20] Sakarcan A Timmons C Seikaly MG. Reversible idiopathic acute renal failure in children with primary nephrotic syndrome. J Pediatr. (1994) 125:723–7. doi: 10.1016/s0022-3476(94)70064-8, PMID: 7965423

[21] Gabriel DP Caramori JT Martim LC Barretti P Balbi AL. High volume peritoneal dialysis vs daily hemodialysis: a randomized, controlled trial in patients with acute kidney injury. Kidney Int Suppl. (2008) 108:S87–93. doi: 10.1038/sj.ki.5002608, PMID: 18379555

[22] Ponce D Balbi A Cullis B. Acute PD: evidence, guidelines, and controversies(☆). Semin Nephrol. (2017) 37:103–12. doi: 10.1016/j.semnephrol.2016.10.011 28153190

[23] Zhou X Dong P Pan J Wang H Xu Z Chen B. Renal replacement therapy modality in critically ill patients with acute kidney injury – a network meta-analysis of randomized controlled trials. J Crit Care. (2021) 64:82–90. doi: 10.1016/j.jcrc.2021.03.011, PMID: 33836397

[24] Tam P. Peritoneal dialysis and preservation of residual renal function. Perit Dial Int. (2009) 29:108–10. doi: 10.1177/089686080902902S20 19270196

[25] Cooper S Iliescu EA Morton AR. The relationship between dialysate protein loss and membrane transport status in peritoneal dialysis patients. Adv Perit Dial. (2001) 17:244–7. PMID: 11510285

[26] Kopanati S Baum M Quan A. Peritoneal protein losses in children with steroid-resistant nephrotic syndrome on continuous-cycler peritoneal dialysis. Pediatr Nephrol. (2006) 21:1013–9. doi: 10.1007/s00467-006-0012-y 16773415

[27] Goldwasser P Feldman JG Barth RH. Serum prealbumin is higher in peritoneal dialysis than in hemodialysis: a meta-analysis. Kidney Int. (2002) 62:276–81. doi: 10.1046/j.1523-1755.2002.00415.x, PMID: 12081589

