# Supplementary material for: Successful treatment of peritoneal dialysis for two patients with refractory nephrotic syndrome and acute kidney injury: a case report
Source: Front Med (Lausanne). 2023 Oct 18;10:1263780. doi: 10.3389/fmed.2023.1263780 (PMC10618994; doi:10.3389/fmed.2023.1263780)
Supplement: Supplementary file 1 [file Table_1.DOCX]

Supplementary Material

| **patient** | **PD treatment weeks** | **scr(mg/dl)** | **proteinuria(g/24h)** | **SAlb(g/L)** | **urinary volume(ml)** |
| --- | --- | --- | --- | --- | --- |
| A | -24 | 1.64 |  | 10.1 |  |
|  | -22 | 3.22 |  | 12 |  |
|  | -21 | 4.07 |  |  |  |
|  | -21 | 2.98 | 28.17 | 17.2 | 1700 |
|  | -20 | 2.63 | 17.04 | 17.9 | 1000 |
|  | -14 | 2.3 | 18.26 | 19.7 | 700 |
|  | -11 | 3.14 | 10.92 | 20.6 | 700 |
|  | -6 | 2.73 | 13.76 | 23 | 700 |
|  | 0 | 3.23 | 16.13 | 22.7 | 1160 |
|  | 2 | 4.82 |  | 21.5 |  |
|  | 6 | 2.96 | 13.4 | 19.8 | 1500 |
|  | 14 | 1.81 | 5.97 | 22.6 | 1500 |
|  | 16 | 1.58 | 4.74 | 27 | 1900 |
|  | 18 | 1.65 | 6.42 | 26.1 | 1800 |
|  | 24 | 1.79 | 6.14 | 25.5 | 2900 |
|  | 30 | 2.26 | 4.2 | 24.8 | 1100 |
|  | 33 | 1.55 | 9.58 | 36.4 | 2100 |
|  | 37 | 1.62 | 9.48 | 30.9 | 1800 |
|  | 47 | 2.01 | 13.33 | 28.9 | 2200 |
|  | 53 | 1.85 | 5.12 | 31.4 | 2800 |
|  | 55 |  | 4.33 |  | 3100 |
|  | 60 | 1.56 | 4.21 | 38 | 2800 |
|  | 65 | 1.78 | 1.77 | 44.3 | 2500 |
|  | 69 | 1.71 | 1.03 | 43.7 | 2000 |
|  | 77 | 1.79 | 0.78 | 47.3 | 2000 |
|  | 89 | 1.78 | 0.73 | 45.7 | 2100 |
|  | 104 | 1.68 | 0.85 | 45.4 | 2300 |

**Supplementary Table 1A.** Follow-up parameters of patient A.

| **patient** | **PD treatment weeks** | **scr(mg/dl)** | **proteinuria(g/24h)** | **SAlb(g/L)** | **urinary volume(ml)** |
| --- | --- | --- | --- | --- | --- |
| B | -18 | 1.58 |  | 30.9 |  |
|  | -13 | 1.64 | 10.48 | 18.2 |  |
|  | -11 | 2.38 |  | 20.8 |  |
|  | -9 | 2.42 | 20.18 | 17.6 | 1400 |
|  | -5 | 2.1 | 18.65 | 18.9 | 1500 |
|  | 0 | 2.25 | 10.55 | 23.6 | 200 |
|  | 7 | 3.11 |  | 27.6 | 350 |
|  | 26 | 2.56 | 8.57 | 27.8 | 800 |
|  | 45 | 2.96 | 6.47 | 35.5 | 1200 |
|  | 58 | 3.12 | 3.75 | 36.9 | 2400 |
|  | 63 | 3.07 | 3.68 | 37.7 | 2100 |
|  | 64 | 3.98 |  | 37.5 | 2000 |
|  | 66 | 4.37 | 1.85 | 38.2 | 1400 |
|  | 70 | 3.15 | 3.69 | 31.1 | 1800 |
|  | 72 | 3.29 | 2.08 | 35.7 | 1900 |
|  | 76 | 2.9 | 1.16 | 38.1 | 1500 |
|  | 80 | 2.52 | 1.9 | 40.5 | 2200 |
|  | 93 | 3.06 | 0.87 | 44 | 1400 |
|  | 104 | 3.05 | 0.85 | 43.3 | 1900 |
|  | 121 | 2.7 | 0.44 | 39.7 | 1400 |

**Supplementary Table 1B.** Follow-up parameters of patient B.
